# Red Blood Cell Parameters, Iron Metabolism and Vitamin B12 Status in Children with Obesity: Associations with Diet and Obesity-Related Complications

**DOI:** 10.3390/nu18101566

**Published:** 2026-05-14

**Authors:** Ewelina Cichocka-Mroczek, Anna Iwańska, Dawid Goncerz, Dorota Łukasik, Aleksandra Molek, Małgorzata Wójcik, Agnieszka Kozioł-Kozakowska

**Affiliations:** 1 Laboratory of Pediatric Dietetics, Department of Pediatrics, Gastroenterology and Nutrition, Jagiellonian University Medical College, 30-663 Cracow, Poland; ewelina.cichocka-mroczek@uj.edu.pl; 2 Doctoral School of Medical and Health Sciences, Jagiellonian University Medical College, 30-663 Cracow, Poland; anna.iwanska@doctoral.uj.edu.pl (A.I.); dawid.goncerz@doctoral.uj.edu.pl (D.G.); 3 Department of Pediatric and Adolescent Endocrinology, Chair of Pediatrics, Pediatric Institute, Jagiellonian University, 30-663 Cracow, Poland; malgorzata.wojcik@uj.edu.pl; 4 Interclinical Center for the Treatment of Childhood Obesity, University Children’s Hospital in Kraków, ul. Wielicka 265, 30-663 Cracow, Poland; 5 Students’ Scientific Group, Department of Paediatric and Adolescents Endocrinology, Chair of Paediatrics, Paediatric Institute, Jagiellonian University Medical College, 30-663 Cracow, Polandaleksandra.molek@gmail.com (A.M.)

**Keywords:** childhood obesity, erythrocyte parameters, iron status, vitamin B12, dietary intake, body composition, adiposity, ferritin, total iron-binding capacity, pediatric population

## Abstract

Aim: To assess abnormalities in red blood cell parameters, iron metabolism, and vitamin B12 status in children with obesity, and to evaluate the influence of dietary intake and obesity-related complications on these variables. Methods: A retrospective analysis was conducted in 152 children with obesity. Anthropometric, biochemical, and hematological parameters were assessed. Dietary intake was evaluated in a subgroup of 33 participants using 3-day food records. Results: No cases of low hemoglobin levels were identified. However, elevated TIBC and occasional low ferritin levels suggested disturbances in iron metabolism. BMI Z-score was positively associated with red blood cell count and selected iron metabolism markers, whereas higher body fat percentage was negatively associated with hemoglobin and hematocrit. Dietary analysis indicated that protein and vitamin B12 intake were associated with erythrocyte parameters, while no associations were found for iron or folate intake. Elevated liver enzymes were associated with higher hemoglobin, hematocrit, and MCV values. Conclusions: Pediatric obesity was not associated with low hemoglobin levels but may be linked to early, subclinical disturbances in iron metabolism. These findings should be confirmed using more comprehensive biomarkers. Dietary factors, particularly vitamin B12 intake, may contribute to variability in erythrocyte parameters; however, these associations should be interpreted with caution. The observed relationship between liver function and erythrocyte indices warrants further investigation.

## 1. Introduction

Childhood obesity has become a major global health problem, characterized not only by excess body fat but also chronic, low-grade inflammation. This inflammatory state contributes to the development of metabolic complications, including insulin resistance and MASLD (metabolic dysfunction-associated steatotic liver disease) [1]. Increasing attention has also been paid to the phenomenon of the “double burden” of obesity and malnutrition, in which excessive caloric intake coexists with deficiencies of essential micronutrients such as vitamin D, iron, and vitamin B12 in pediatric populations [2,3]. Despite excessive energy intake, children with obesity frequently present with micronutrient deficiencies, which may have a pathogenic role in obesity-related metabolic comorbidities [4]. Among these, disturbances in iron metabolism appear particularly important. A large meta-analysis demonstrated that obesity is associated with lower serum iron levels and reduced transferrin saturation [5]. Recent global data (2025) further indicate that anemia is more common in children with obesity, with low hemoglobin levels observed in 22.6% compared to 10.9% in normal-weight controls [6]. Similarly, data from Romania indicate that iron deficiency may affect up to 50% of overweight children, with 38.5% developing iron deficiency anemia [3]. Overall, the pooled prevalence of iron deficiency in children with obesity is estimated at approximately 20.07% [7]. Early adolescence (10–13 years) appears to be a particularly vulnerable period, with nearly a threefold higher risk of anemia compared to peers [8]. The underlying mechanism is thought to involve chronic inflammation related to obesity, which stimulates hepatic production of hepcidin, a key regulator of iron homeostasis. Elevated hepcidin levels inhibit intestinal iron absorption and reduce iron release from macrophages, leading to functional iron deficiency and impaired erythropoiesis [1,5]. This may also explain the reduced effectiveness of iron supplementation observed in this population [9]. In addition to iron, other micronutrients may also be involved. Recent studies have shown an inverse relationship between serum vitamin B12 levels and adiposity in children and adolescents, suggesting a higher risk of deficiency in obese individuals [10]. A high prevalence of vitamin D deficiency has also been described, with a relative risk of 1.41 compared to normal-weight peers [11]. However, evidence remains inconsistent; for example, a large Polish cohort study of 500 children with obesity did not confirm vitamin B12 deficiency or an association between obesity severity and B12 levels [12]. Despite these findings, the relationship between dietary intake, iron metabolism, and erythrocyte parameters in children with obesity remains unclear. In particular, it is not well established how nutritional patterns and obesity-related metabolic complications jointly influence hematological indices, iron status, and vitamin B12 levels in this population. Therefore, the aim of this study was to assess the prevalence of abnormalities in red blood cell parameters, iron metabolism, and vitamin B12 status in children with obesity, and to evaluate dietary intake and influence of dietary intake and obesity-related complications on these variables.

## 2. Material and Methods

The study was conducted in a cohort of children with obesity treated at the University Children’s Hospital in Kraków. A total of 152 children with obesity were included; 55.3% were boys. Mean age was 13.2 years (SD 3.5) and mean BMI Z-score was 2.26 (SD 0.41; range 1.08–3.84).

The analysis included hematological parameters (RBC—red blood cell count, Hb—hemoglobin, HCT—hematocrit, MCV—mean corpuscular volume), markers of iron metabolism (TIBC—total iron-binding capacity, ferritin), serum vitamin B12 levels as well as inflammatory markers, including *C*-reactive protein (CRP) and white blood cell count (WBC).

CRP and WBC were used to assess the presence of inflammation. Participants were classified as having positive inflammatory markers if CRP and/or WBC exceeded predefined thresholds. Additionally, relationships between blood parameters, obesity severity, dietary intake, and obesity-related complications were assessed. The severity of obesity was evaluated using BMI Z-score and body fat percentage measured by bioimpedance analysis. Dietary intake was analyzed in a subset of 33 participants (21.7%), based on available 3-day food records. The reduced number of analyzed dietary records resulted from several factors. In 13 cases (8.6%), the dietary records contained substantial missing quantitative data (e.g., incomplete portion sizes or amounts), which made reliable nutritional analysis impossible. An additional 24 participants (15.8%) provided dietary records that had incomplete quantitative information (e.g., only food items were reported without portion sizes or weights), and therefore their data could not be included in the analysis. The largest group consisted of participants who did not submit their dietary records to the dietitian (*n* = 82; 53.9% of the study population).

Therefore, the analysis was conducted using only complete and interpretable records, ensuring reliable results despite the reduced sample size.

Dietary data included intake of energy, carbohydrates, fats, protein per kilogram of body weight, iron, vitamin B12, and vitamin B9, and vitamin C as well as selected dietary ratios (iron-to-calcium and iron-to-vitamin C). Normality of distribution was assessed using the Shapiro–Wilk test (*p* > 0.05 was considered indicative of normal distribution) for the full study group (*n* = 152). In subgroups with fewer than 30 observations, normality was evaluated using skewness and kurtosis (acceptable range: −1 to 1 and −2 to 2, respectively). Since at least one variable in each analyzed pair showed a non-normal distribution, non-parametric methods were applied throughout.

For between-group comparisons, the Mann–Whitney U test was used in all comparisons, including subgroups where normality criteria were met, due to marked group imbalance and small subgroup size (e.g., elevated liver enzymes: *n* = 10). Correlations were assessed using Spearman’s rank correlation coefficient. To assess the potential impact of inflammation on red blood cell parameters, participants were categorized based on the presence of inflammatory markers (elevated CRP and/or WBC). Comparisons of RBC, hemoglobin (Hb), and hematocrit (Hct) between groups with and without inflammatory markers were performed using the Mann–Whitney U test. An additional analysis was conducted for isolated CRP elevation (>10 mg/L).

To evaluate the relationship between inflammation and iron status, correlations between ferritin and inflammatory markers (CRP and WBC) were assessed using Spearman’s rank correlation coefficient.

Statistical significance was set at *p* < 0.05. Statistical analyses were performed using SPSS 11.

Laboratory parameters were interpreted according to age- and sex-specific reference ranges provided by the local laboratory. The following cut-offs were applied to identify values outside the normal range, based on local laboratory references: hemoglobin (Hb) < 10.6 g/dL; ferritin < 7 µg/L in children aged 6 months–15 years, <5 µg/L in females > 15 years, and <15 µg/L in males > 15 years; vitamin B12 < 200 pg/mL; total iron-binding capacity (TIBC) > 70 μmol/L; and vitamin D (25(OH)D) < 20 ng/mL. Values below these cut-offs were considered low, and values above were considered elevated, without assigning clinical diagnoses of anemia or micronutrient deficiency. Elevated CRP was defined as >10 mg/L. Elevated WBC was defined as >11.0 × 10^3^/µL in boys and >11.4 × 10^3^/µL in girls. Notably, a single hemoglobin cut-off (<10.6 g/dL) was applied across all age and sex groups based on local laboratory standards.

The study was conducted following approval from the Ethics Committee of the Jagiellonian University Medical College (approval nr 118.0043.1.284.2025).

## 3. Results

Selected hematological and biochemical abnormalities are summarized in Table 1. None of the participants met the criteria for low hemoglobin level (Hb < 10.6 g/dL). However, selected abnormalities in iron metabolism and vitamin status were observed (Table 1). Elevated TIBC was observed in over one-third of the cohort whereas ferritin levels below the lower reference limit and vitamin B12 concentrations below the reference range were relatively rare. In contrast, vitamin D levels below the reference range were observed in half of the study population.

Elevated CRP (>10 mg/L) was observed in 11 of 152 participants (7.2%), while elevated WBC was identified in five participants (3.3%). Concurrent elevation of both CRP and WBC occurred in one participant (0.6%). Overall, 15 participants (9.9%) had at least one elevated inflammatory marker (CRP and/or WBC).

No statistically significant differences were observed in RBC, hemoglobin (Hb), or hematocrit (Hct) between participants with and without elevated inflammatory markers (RBC: *p* = 0.263; Hb: *p* = 0.07; Hct: *p* = 0.424).

In the analysis comparing participants with elevated CRP (>10 mg/L) to those with normal CRP levels, a statistically significant difference in hemoglobin concentration was observed (*p* = 0.035). Median hemoglobin was 14.1 g/dL (IQR: 1.6) in the normal CRP group and 13.2 g/dL (IQR: 1.2) in the elevated CRP group. No significant differences were found for RBC (*p* = 0.171) or Hct (*p* = 0.189).

The relationship between obesity severity and hematological parameters are presented in Table 2. BMI Z-score showed statistically significant positive correlations with RBC, TIBC, and ferritin. Although these correlations were weak to moderate in strength, they were consistent across the analyzed parameters. These findings indicate an association between higher BMI Z-score and these hematological and iron-related indices; however, causal or mechanistic interpretations cannot be inferred from these data.

No significant associations were found between BMI Z-score and Hb, HCT, or vitamin B12 levels. In contrast, body fat percentage measured by bioimpedance demonstrated a different pattern of associations (Table 2). Significant negative correlations were observed for Hb and HCT, indicating that higher adiposity may be associated with lower values of these parameters. No statistically significant relationships were found between body fat percentage and RBC, TIBC, ferritin, or vitamin B12.

Overall, BMI Z-score was associated with multiple positive correlations involving erythrocyte count and iron metabolism markers, whereas higher body fat percentage was linked to lower hemoglobin and hematocrit values. Detailed correlations between obesity indices and hematological parameters are provided in the Supplementary Materials (Table S2).

To address potential confounding by sex, additional sex-stratified analyses were performed. Distinct patterns of associations were observed between boys and girls. A significant positive correlation between BMI Z-score and RBC was found only in girls (rho = 0.288, *p* = 0.018), whereas no such association was observed in boys (*p* = 0.506). In contrast, body fat percentage was negatively correlated with hemoglobin and hematocrit exclusively in boys (Hb: rho = −0.421, *p* < 0.001; HCT: rho = −0.404, *p* < 0.001), while these relationships were not significant in girls (Hb: *p* = 0.445; HCT: *p* = 0.053). Furthermore, a positive correlation between BMI Z-score and TIBC was identified only in girls (rho = 0.252, *p* = 0.040), with no association observed in boys (*p* = 0.560). Notably, the previously observed correlation between BMI Z-score and ferritin was no longer significant after sex stratification (girls: *p* = 0.693; boys: *p* = 0.342).

Analyses stratified by age were not performed, as no clinically justified cut-off could be identified for dividing the cohort, given the substantial inter-individual variability in the timing of pubertal onset. Importantly, data on pubertal status according to Tanner staging were not available in the dataset, which precluded more precise adjustment for the developmental stage.

Exploratory dietary associations based on the subgroup analysis (*n* = 33) are summarized in Table 3. Protein intake showed a significant positive correlation with RBC, while protein intake per kilogram of body weight was positively associated with serum vitamin B12 concentration. Furthermore, dietary vitamin B12 intake was positively correlated with both RBC and ferritin levels. These exploratory associations observed in a small subgroup (*n* = 33) suggest a potential relationship between protein and vitamin B12 intake and selected hematological parameters; however, these findings should be interpreted with caution due to the limited sample size and potential selection bias related to missing dietary data. However, given the exploratory nature of this analysis and the limited subgroup size, these findings should be interpreted cautiously and considered hypothesis-generating rather than confirmatory.

No significant correlations were observed for hemoglobin, hematocrit, TIBC, MCV, dietary iron, vitamin B9, or the iron-to-vitamin C ratio.

The analysis of obesity-related complications included hyperinsulinism, impaired fasting glucose (IFG), and elevated liver enzymes. Due to the very high prevalence of vitamin D deficiency, this parameter was excluded from further analysis. Impaired glucose tolerance (IGT) was also excluded due to insufficient sample size. No significant differences in hematological parameters were observed between patients with and without hyperinsulinism (*p* > 0.05 for all comparisons). Due to the small number of patients with IFG (*n* = 6), non-parametric testing (Mann–Whitney U test) was applied; however, no statistically significant differences were identified between groups (*p* > 0.05). In contrast, patients with elevated liver enzymes (*n* = 9) demonstrated significantly higher values of selected red blood cell parameters compared to those with normal enzyme levels. Median hemoglobin was higher in the elevated enzyme group (14.7 vs. 13.8 g/dL; *p* = 0.009), as was hematocrit (44.05 vs. 41.1%; *p* = 0.01) and MCV (87 vs. 83.75 fL; *p* = 0.048).

The subgroup consisted of 33 children and adolescents, including 17 (51.5%) females and 16 (48.5%) males. The mean age of participants was 13.5 ± 2.8 years (range: 9.0–17.98 years), with a mean BMI Z-score of 2.24 ± 0.45 (range: 1.12–2.92). It should be noted that dietary data were available only for a subset of participants, and the pattern of missing data may introduce selection bias, potentially limiting the generalizability of these findings.

Fe RDA children 7–9 y.o: 10 mg/d; boys: 10–12 y.o. 10 mg/d, 13–18 2.4 µg/d; girls 10–12 y.o. 10 mg/d; 13–15 y.o.: 12 mg/d/Vit B12 RDA children 7–12 y.o. 1.8 µg/d; 13–18 y.o. 2.4 µg/d/Vit B9 RDA children 7–12 y.o. 200 µg/d; 13–18 y.o. 400 µg/d/ Vit C RDA: children 7–12 y.o. 50 mg/d; boys 13–18 y.o. 75 mg/d; girls 13–18 y.o. 65 mg/d.

Energy intake was frequently below estimated requirements based on ideal body weight, with most participants not reaching 100% of the recommended intake. This observation should be interpreted in the context of known limitations of self-reported dietary assessment. In several cases, reported energy intake was extremely low (<50% of requirements). Protein intake, particularly when expressed per ideal body weight, was generally elevated. Most participants exceeded 100% of the recommended intake, with some reaching values above 200–300%. In contrast, protein intake calculated per actual body weight was lower, reflecting excess adiposity in the study group. Fat and carbohydrate intake showed considerable inter-individual variability, with no consistent dietary pattern observed across the group.

Iron intake exceeded RDA values in 36.4% of participants (*n* = 12), suggesting that while a subset of the group had adequate or high intake, the majority did not meet recommended levels. Folate (vitamin B9) intake was insufficient in a large proportion of participants. As many as 78.8% (*n* = 26) failed to meet 100% of the RDA, highlighting a consistent and significant inadequacy in folate intake across the group. Vitamin B12 intake was generally adequate or high, with 72.7% (*n* = 24) of participants meeting or exceeding RDA values. Vitamin C intake demonstrated marked variability. While 57.6% (*n* = 19) of participants met or exceeded recommendations, a substantial proportion (42.4%, *n* = 14) had intake below recommended levels. More detailed nutritional data are provided in the Supplementary Materials (Table S1).

## 4. Discussion

The present study evaluated hematological parameters and iron metabolism in pediatric patients with obesity. Despite significant adiposity, none of the participants presented with hemoglobin levels below the lower reference limit, suggesting that overt anemia may not be a common feature in this cohort. Notably, this finding differs from previous reports, including a recent study by Trabzon et al. that described an anemia prevalence of 22.6% in children with obesity [6]. Several factors may account for this discrepancy, including differences in anemia definitions, hemoglobin cut-off values, population characteristics (age, sex, degree of obesity), and study design.

Therefore, these findings should be interpreted with caution, and direct comparisons across studies require consideration of these factors.

Although previous meta-analyses have reported an increased risk of iron deficiency in this population [5], our findings indicate that clinically apparent disturbances may be absent.

Nevertheless, elevated TIBC was observed in a substantial proportion of patients, while low ferritin levels were rare. This pattern suggests early dysregulation of iron metabolism, potentially preceding overt abnormalities in hemoglobin. The results highlight an important distinction between total body mass and adiposity. BMI Z-score was positively associated with RBC, TIBC, and ferritin levels, which may reflect increased physiological demand as well as the role of ferritin as an acute-phase reactant in obesity-related inflammation [1]. Similar observations have been reported in pediatric populations, where higher RBCs were noted in children with obesity [13]. At the same time, the interpretation of ferritin is limited, as inflammation may mask underlying disturbances in iron status [1]. In contrast, a higher body fat percentage was associated with lower hemoglobin and hematocrit values. The opposing directions of associations observed for BMI Z-score and body fat percentage may reflect the well-recognized limitations of BMI as a proxy for metabolic health. BMI does not distinguish between lean and fat mass or reflect fat distribution, whereas body fat percentage may more accurately capture adiposity-related metabolic alterations. This interpretation is consistent with growing evidence that BMI alone does not adequately reflect metabolic risk and recent consensus statements have emphasized the need for multidimensional assessment of obesity-related health status [14].

These findings support the concept that adiposity itself may influence erythropoiesis, potentially through low-grade inflammation and hepcidin-mediated mechanisms [1,9,15]. However, this interpretation remains speculative in the absence of direct inflammatory or regulatory markers (e.g., hepcidin). Consequently, iron may become functionally unavailable despite normal or elevated ferritin concentrations.

This potential interaction between inflammation and erythropoiesis is further supported by the observed relationship between CRP and hemoglobin.

Although a statistically significant difference in hemoglobin concentration was observed between children with elevated CRP (>10 mg/L) and those with normal CRP, hemoglobin values in both groups remained within the reference range, and no participant met the diagnostic criteria for anemia. These findings suggest that CRP elevation may be associated with a modest reduction in hemoglobin; however, the clinical relevance of this difference appears limited in the present cohort.

This observation may suggest a potential relationship between adiposity and altered erythropoietic parameters; however, the underlying mechanisms cannot be determined based on the present data.

Metabolic complications of obesity showed a heterogeneous relationship with hematological parameters. No significant associations were observed for hyperinsulinemia or impaired fasting glucose, suggesting that early disturbances in glucose metabolism may not substantially influence erythropoiesis.

Previous studies have reported inconsistent findings, with some suggesting an increased prevalence of anemia in individuals with IFG [16], while others indicate that anemia is more strongly associated with advanced metabolic disease [17]. Additionally, iron deficiency has been shown to influence HbA1c independently of glycemia, further complicating the relationship between iron metabolism and glucose homeostasis [18]. In contrast, elevated liver enzymes were associated with higher hemoglobin, hematocrit, and MCV values, suggesting a potential role of hepatic function in modulating red blood cell parameters. However, these findings were derived from a very small subgroup (*n* = 9). Therefore, they should be interpreted with caution and considered a preliminary, exploratory association rather than a stable clinical pattern.

The liver is central to iron regulation through hepcidin production, which is increased in obesity-related inflammation [9]. This mechanism has been proposed to contribute to altered iron distribution and availability [14,19]; however, it cannot be confirmed in the present study.

Moreover, liver dysfunction is closely linked to metabolic disturbances such as insulin resistance and prediabetes [20], which may further influence hematological parameters. The observed increase in MCV may also reflect subtle alterations in erythrocyte metabolism or vitamin handling, even in the absence of overt deficiencies [21].

Dietary analysis indicated that protein and vitamin B12 intake were the main nutritional factors associated with hematological parameters. Both were positively associated with RBC and ferritin levels, suggesting their relevance for erythropoiesis [22].

This relationship is biologically plausible, as protein-rich foods constitute the main dietary source of vitamin B12, which is essential for red blood cell maturation [23,24,25].

In contrast, no association was observed for iron intake or vitamin B9, supporting the concept that obesity-related inflammation may impair iron utilization independently of dietary intake [5,26,27]. Additionally, reduced micronutrient bioavailability has been described in obesity [28]. Taken together, these findings suggest that pediatric obesity is associated with early, subclinical disturbances in iron metabolism rather than overt anemia. These alterations appear to be more closely related to adiposity and inflammation than to total body mass or dietary iron intake. This supports the concept of “hidden malnutrition” in obesity, in which excess energy intake coexists with qualitative nutritional deficiencies [29].

However, these results should be interpreted with caution, as dietary analysis was performed in only 33 out of 152 participants, which substantially limits statistical power and the generalizability of these findings. In addition, the lack of association for iron intake may reflect the small sample size and potential measurement error in dietary assessment rather than a true absence of biological relevance. In addition, the lack of association for iron intake may reflect the small sample size and potential measurement error in dietary assessment rather than a true absence of biological relevance.

A high prevalence of underreporting of energy intake was observed, consistent with the previous literature in individuals with obesity [30]. Therefore, dietary intake data should be interpreted with caution. This limitation likely affects estimates of micronutrient intake, particularly for nutrients derived from fruits and vegetables such as folate and vitamin C, and may partly explain the lack of associations between iron intake and hematological parameters.

Despite this limitation, protein intake was frequently excessive, particularly when expressed relative to ideal body weight. Given its role in erythropoiesis and its association with vitamin B12 intake, this dietary pattern may contribute to the observed relationships with RBC parameters.

Folate (vitamin B9) intake was insufficient in 78.8% (*n* = 26) of participants, while over 40% did not meet recommended vitamin C intake levels. This variability may have contributed to the lack of clear associations between dietary intake and iron-related parameters, particularly given the role of vitamin C in non-heme iron absorption.

Nevertheless, the high prevalence of inadequate folate and vitamin C intake should be interpreted with caution, as both nutrients are derived largely from foods prone to omission or misreporting in dietary records, such as fruits and vegetables. Therefore, their intake may be underestimated.

In contrast, vitamin B12 intake appeared adequate in most participants, likely reflecting more consistent reporting of animal-derived products. This may partly explain the observed associations between protein and vitamin B12 intake and hematological parameters, showing positive relationships with RBC and ferritin levels [22]. Given that animal protein is the primary dietary source of vitamin B12 [23], these associations are biologically plausible. Considering the role of vitamin B12 in erythropoiesis, even subtle variations in intake may influence hematological indices [24,25]. Overall, these findings suggest that pediatric obesity is associated with early, subclinical disturbances in iron metabolism rather than overt anemia. These alterations appear to be more closely related to adiposity and inflammation than to total body mass or dietary iron intake. This supports the concept of “hidden malnutrition” in obesity, in which excess energy intake coexists with qualitative nutritional deficiencies [29].

The results should be interpreted in the context of the study design, particularly considering the absence of a control group, which could provide additional comparative insight into the observed outcomes and further strengthen the interpretation of the findings.

### Limitations

This study has several limitations that should be considered when interpreting the results. First, the retrospective design limits the ability to establish causal relationships between obesity, dietary intake, and hematological parameters. Second, the dietary analysis was performed in only a subset of participants (*n* = 33), which may introduce selection bias and substantially limit the statistical power and generalizability of the observed associations. Third, one of key markers of iron metabolism and inflammation, hepcidin, was not assessed, which limits the ability to fully interpret the mechanisms underlying the observed alterations in iron homeostasis. Importantly, in the absence of concurrent inflammatory marker, ferritin values in this cohort cannot be reliably interpreted as accurate indicators of iron stores, as they may be influenced by obesity-related low-grade inflammation.

Additionally, the definition of anemia based on a single hemoglobin cut-off value may not fully reflect age- and sex-specific diagnostic criteria, potentially leading to underestimation of anemia prevalence. Furthermore, ferritin, used as a marker of iron stores, may be influenced by low-grade inflammation associated with obesity, which could mask underlying iron deficiency. In addition, the study was conducted without a control group, which limits the comparative interpretation of the observed findings. The inclusion of an appropriately matched control group in future studies could provide additional context for the evaluation of the reported outcomes.

Finally, the relatively small sample sizes in some subgroup analyses, particularly those related to dietary intake and obesity-related complications, may limit the robustness of these findings and should be interpreted with caution. Moreover, dietary assessment based on self-reported records is prone to systematic under-reporting of energy and nutrient intake, particularly in individuals with obesity, which may bias observed associations.

## 5. Conclusions

Pediatric obesity in this cohort was not associated with low hemoglobin levels but may be linked to early alterations in iron-related parameters. These findings suggest that changes in iron homeostasis could precede overt hematological abnormalities; however, given the limited set of biomarkers and the absence of key indicators such as hepcidin, serum iron, transferrin saturation, and a control group, these observations should be interpreted with caution and require confirmation in studies using more comprehensive assessments of iron status.

The opposing associations of BMI Z-score and body fat percentage with erythrocyte parameters emphasize the importance of distinguishing between overall body mass and adiposity in clinical evaluation. These findings suggest that BMI alone may not adequately reflect adiposity-related alterations in erythrocyte parameters, and that direct measures of body fat may provide additional clinically relevant information when evaluating hematological status in children with obesity.

Dietary factors, particularly vitamin B12 and protein intake, may contribute to variability in erythrocyte parameters, although these associations require confirmation in larger, well-characterized cohorts.

The observed relationship between elevated liver enzymes and higher erythrocyte indices points to a potential link between hepatic function and erythropoiesis, warranting further investigation.

## Figures and Tables

**Table 1:** Prevalence of selected abnormalities (*n* = 152).

Parameter	*n* (%)
Low hemoglobin level (Hb < 10.6 g/dL)	0 (0%)
Elevated TIBC (>70 μmol/L)	56 (37.0%)
Low Ferritin level *	4 (2.6%)
Low Vitamin D level (<20 ng/mL)	76 (50.0%)
Low B12 level (<200 pg/mL)	5 (3.3%)

* Ferritin below 7 µg/L in children 6 months–15 years, below 5 µg/L in females > 15 years, and below 15 µg/L in males > 15 years.

**Table 2:** Correlations between obesity indices and hematological parameters.

Parameter	BMI Z-Score (*r*, *p*-Value)	Body Fat % (*r*, *p*-Value)
**RBC**	0.305, *p* < 0.001	NS
**Hb**	NS	−0.237, *p* = 0.004
**HCT**	NS	−0.177, *p* = 0.032
**TIBC**	0.173, *p* = 0.034	NS
**Ferritin**	0.167, *p* = 0.041	NS
**Vitamin B12**	NS	NS

NS—not significant (*p* > 0.05).

**Table 3:** Correlations between dietary intake and hematological parameters (*n* = 33).

Dietary Variable	Parameter	Rho	*p*-Value
**Protein**	RBC	0.366	0.039
**Protein/kg BW**	Vitamin B12	0.397	0.022
**Vitamin B12 intake**	RBC	0.375	0.034
**Vitamin B12 intake**	Ferritin	0.374	0.032

BW—body weight.

## Data Availability

The original contributions presented in this study are included in the article/Supplementary Materials. Further inquiries can be directed to the corresponding author(s).

## Supplementary Materials

The following supporting information can be downloaded at: https://www.mdpi.com/article/10.3390/nu18101566/s1. Table S1: Characteristics of macronutrient and micronutrient intake as percentage of recommended daily intake (RDA or IBW/ABW); Table S2: Spearman correlation matrix between obesity indices and hematological parameters.
