# Supplementary material for: Red Blood Cell Parameters, Iron Metabolism and Vitamin B12 Status in Children with Obesity: Associations with Diet and Obesity-Related Complications
Source: Nutrients. 2026 May 14;18(10):1566. doi: 10.3390/nu18101566 (PMC13209332; doi:10.3390/nu18101566)
Supplement: Supplementary file 1 [file nutrients-18-01566-s001.zip › nutrients-4272898-supplementary.pdf]

Table S1: Characteristics of macronutrient and micronutrient intake as percentage of recommended daily intake (RDA or IBW/ABW).

N	Fe %RDA	Vit.C %RDA	Vit. B9 % RDA	Vit. B12 %RDA
1	107	171	77	146
2	87	169	89	138
3	129	155	103	175
4	78	80	57	198
5	134	534	172	265
6	91	351	92	108
7	105	123	82	132
8	100	112	77	139
9	72	52	59	105
10	70	130	69	83
11	52	43	54	65
12	34	26	34	79
13	82	75	49	144
14	52	176	70	110
15	78	162	77	142
16	120	586	135	140
17	67	23	56	122
18	59	42	51	78
19	39	81	36	35
20	61	113	60	86
21	40	22	31	59
22	118	174	102	250
23	103	112	75	139
24	92	138	89	138
25	118	74	88	224
26	56	219	83	190
27	18	97	29	57
28	103	384	101	102
29	58	25	34	155
30	58	29	34	155
31	53	33	49	85
32	106	185	102	100
33	120	233	115	124

Fe % RDA– iron intake % of Recommended Dietary Allowance, vit. C/B9/B12 % RDA– vitamin intake % RDA.

Table S2: Spearman correlation matrix between obesity indices and hematological parameters

Variable	BMI Z-score	Body fat (%)	RBC	Hb	HCT	TIBC	Ferritin
**BMI Z-score**	—	0.499***	0.305***	0.046	0.077	0.173*	0.167*
**Body fat (%)**	0.499***	—	-0.085	-0.237**	-0.177*	0.069	-0.019
**RBC**	0.305***	-0.085	—	0.655***	0.732***	0.173*	0.210*
**Hb**	0.046	-0.237**	0.655***	—	0.930***	-0.095	0.403***
**HCT**	0.077	-0.177*	0.732***	0.930***	—	-0.031	0.322***
**TIBC**	0.173*	0.069	0.173*	-0.095	-0.031	—	-0.317***
**Ferritin**	0.167*	-0.019	0.210*	0.403***	0.322***	-0.317***	—

Values are Spearman's rho coefficients.

- $p < 0.05$, ** $p < 0.01$, *** $p < 0.001$.

RBC – red blood cells; Hb – hemoglobin; HCT – hematocrit; TIBC – total iron-binding capacity.
